# A multivariate soil temperature interval forecasting method for precision regulation of plant growth environment

**DOI:** 10.3389/fpls.2024.1460654

**Published:** 2024-12-26

**Authors:** Hang Yin, Zeyu Wu, Zurui Huang, Yiting Luo, Xiaohan Liu, Xiaojiang Peng, Qiang Li

**Affiliations:** College of Big Data and Internet, Shenzhen Technology University, Shenzhen, China

**Keywords:** soil temperature forecasting, multivariate forecasting, N-HiTS, Gaussian likelihood, multi-objective optimization, interval forecasting

## Abstract

Foliage plants have strict requirements for their growing environment, and timely and accurate soil temperature forecasts are crucial for their growth and health. Soil temperature exhibits by its non-linear variations, time lags, and coupling with multiple variables, making precise short-term multi-step forecasts challenging. To address this issue, this study proposes a multivariate forecasting method suitable for soil temperature forecasting. Initially, the influence of various environmental factors on soil temperature is analyzed using the gradient boosting tree model, and key environmental factors are selected for multivariate forecasting. Concurrently, a point and interval forecasting model combining the Neural Hierarchical Interpolation for Time Series Forecasting (N-HiTS) and Gaussian likelihood function is proposed, providing stable soil temperature forecasting for the next 20 to 120 minutes. Finally, a multi-objective optimization algorithm is employed to search for optimal initial parameters to ensure the best performance of the forecasting model. Experiments have demonstrated that the proposed model outperforms common models in predictive performance. Compared to Long Short-Term Memory (LSTM) model, the proposed model reduces the Mean Absolute Error (MAE) for forecasting soil temperatures over the next 20, 60, and 120 minutes by 0.065, 0.138, and 0.125, respectively. Moreover, the model can output stable forecasting intervals, effectively mitigating the instability associated with multi-step point forecasts. This research provides a scientific method for precise regulation and disaster early warning in facility cultivation environments.

## 1 Introduction

Foliage plants are cultivated primarily for their ornamental qualities, particularly the unique forms, colors, and textures of their leaves. These plants are valued not only for their aesthetic appeal but also for their ability to purify the air and promote emotional well-being (Han and Li-Wen, 2020; Kim et al., 2020). Consequently, they hold significant economic and practical potential. Foliage plants like Aglaonema require a precise growth environment. Their growth halts if the soil temperature deviates from 25-30°C during the day or 20-25°C at night (Lin, 2023). Thus, maintaining environmental parameters within a suitable and stable range is crucial for plant growth, as it can significantly enhance the quality of foliage plants in facility cultivation environments. Among numerous environmental factors, soil temperature (ST) stands out as a pivotal indicator (Chaipong, 2020). It significantly influences key aspects of plant development, such as seed germination, root growth, and the maturation of stems and leaves. Moreover, it is intricately associated with soil moisture levels, microbial activity, and the transformation of organic matter (Myster and Roar, 1995; Heinze et al., 2017; Noia et al., 2018). Therefore, establishing a precise ST forecasting model holds significant value for the management and regulating of plant growth, health, and irrigation practices (Tsai et al., 2020).

ST variations exhibit non-linearity and temporal lag, influenced by various environmental factors such as air temperature, air humidity, soil moisture, and solar radiation. Therefore, forecasting ST is a typical multivariate time series forecasting task. Soil temperature forecasting mainly includes three developmental stages: mechanistic models, machine learning, and deep learning. Early experts established some mechanistic models such as dynamic model (Kıyan et al., 2013; Joudi and Farhan, 2015) and thermodynamic model (Ali et al., 2020) for predicting greenhouse environmental factors. However, these methods are susceptible to external conditions and parameter settings, making them unstable. With the rapid development of Internet of Things (IoT) technology, various data-driven models based on machine learning, including Random Forests (Hamrani et al., 2020), Support Vector Regression (Yu et al., 2016), Seasonal Autoregressive Integrated Moving Average (Zeynoddin et al., 2020), Artificial Neural Networks (Petrakis et al., 2022), have been employed for short-term single-step forecasting studies of greenhouse environmental factors. However, the cultivation of foliage plants is a ongoing endeavor, and single-step forecasts cannot fully meet the needs of precise environmental regulation. Therefore, there is a pressing need for research focused on multi-step forecasting. In recent years, the emergence of deep learning models has presented new opportunities to address the challenge of multivariate multi-step forecasting in greenhouse environments. Liu et al. (2022) combined the Long Short-Term Memory (LSTM) model with multiple environmental variables to preliminarily achieve multi-step forecasting of ST. He et al. (2022) tested the potential the Gated Recurrent Unit (GRU) model in multivariate multi-step forecasting within greenhouse environmental factors, affirming the GRU model outperforms machine learning models. However, the environmental factors in a greenhouse are highly interrelated and complex, and a single recurrent neural network lacks the capacity to effectively capture the dependencies within high-dimensional data (Javed et al., 2022). In response to this challenge, some researchers (Jin et al., 2021; Yang et al., 2023; Li et al., 2024) introduced attention mechanisms to conduct deeper feature extraction on multivariate environmental data, thereby further enhancing the multi-step forecasting performance of baseline models for environmental factors, but attention mechanisms also increased the inference time and the risk of overfitting (Liu et al., 2024). Besides, Shi et al. (2024) integrated convolutional neural networks (CNN), LSTM, and the sparrow search algorithm (SSA) to establish a greenhouse environment forecasting model, achieving more precise results compared to individual models. However, this approach also led to increased inference time and a larger model size

Although past research has made significant progress in improving model accuracy, there are limitations in model stability and real-time performance: i) While stacking models and integrating attention mechanisms can improve predictive performance, these enhancements typically come at the expense of increased model complexity and longer training time, posing challenges for real-time inference and edge deployment. ii) In relatively enclosed environments of facility cultivation, soil temperature variations are sensitive to microclimate changes induced by sunlight, temperature fluctuations, and human activities, often exhibiting short-term fluctuations, which indicates that multi-step point forecasts inevitably contain errors, introducing uncertainty into decision-making processes. iii) Determining the hyperparameters of neural networks presents challenges. Common tuning methods, such as grid search, have computational burdens and time requirements that grow exponentially with the number of hyperparameters, making it tough to determine them quickly.

To alleviate these challenges, this study aims to propose an efficient and swift method for short-term multi-step forecasting, which is intended for precise ST management and dynamic regulation in the cultivation process of foliage plants. It innovatively integrates multivariate forecasting, interval forecasting, and multi-objective optimization algorithms. The specific contributions of this research are as follows:

This study focus on multivariate time series forecasting and introduces a feature analysis framework based on gradient boosting tree models. The goal is to enhance the accuracy and stability of ST forecasting by integrating various environmental factors. Key characteristics of this method include its straightforward operation, efficient execution speed, and its ability to perform objective and comprehensive feature extraction.
In response to the issues of model over-complexity and the risk of multi-step forecasting errors, this study establishes the N-HiTS-G model for point and interval forecasting of soil temperatures. This model combines the neural hierarchical interpolation for time series forecasting methodology with Gaussian likelihood function, effectively enhancing the precision and stability of model. Compared to existing models based on RNNs and attention mechanisms, the proposed model not only achieves faster inference speed and higher forecasting accuracy but also produces stable forecasting intervals, enhancing its suitability for deployment and utilization in foliage plant facility cultivation environments.
To reduce the time and labor costs associated with finding the optimal hyperparameters for the model, this study utilizes the multi-objective optimization algorithm to optimize the parameters of the model and further proposes the SP-N-HiTS-G model. Multi-objective optimization algorithm not only ensures the effectiveness of point and interval forecasting but also thoroughly explores the predictive performance of the model. The result indicates that the SP-N-HiTS-G model achieves more precise and stable multi-step forecasting of soil temperature in facility cultivation environments.

## 2 Methodology

### 2.1 Soil temperature forecasting framework

**Figure 1:**
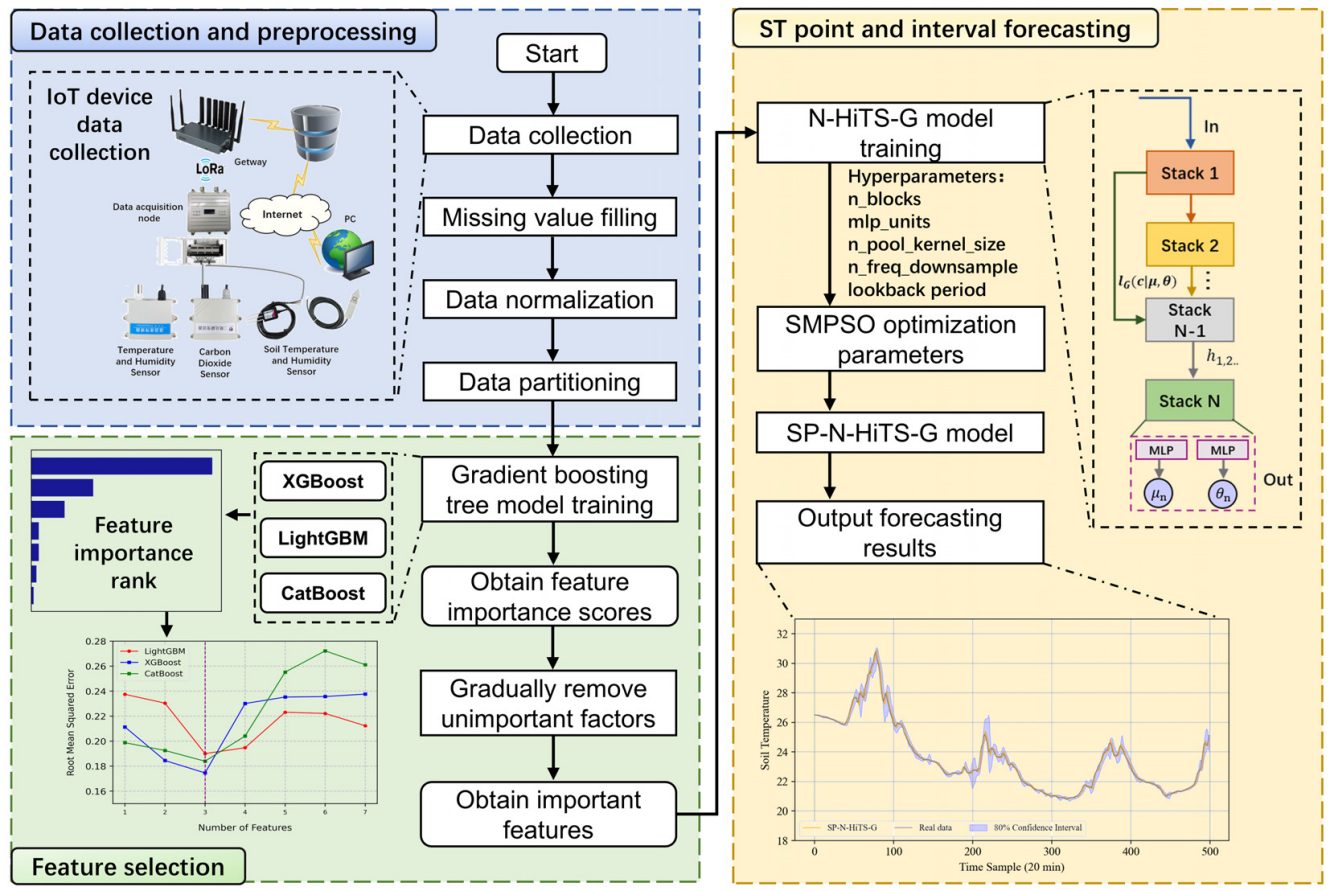
Short-term multi-step forecasting method architecture for soil temperature.

The architectural overview of the ST forecasting method proposed in this study primarily consists of three modules: data acquisition and preprocessing, selection of critical environmental factors, and forecasting of soil temperature. The initial module predominantly involves the utilization of various IoT sensors to gather environmental data within the greenhouse, and supplemented by employing linear interpolation techniques to address missing values, thus ensuring data integrity. The second module serves as a feature selection component, wherein we employ advanced gradient boosting tree models including XGBoost, LightGBM, and CatBoost to analyze and select important environmental factors pertinent to ST. This aids in reducing training time and enhancing predictive accuracy, thereby elevating model performance. In the third module, we establish a novel forecasting model based on SP-N-HiTS-G, which features high accuracy, rapid inference, and the ability to generate forecasting intervals. This model can reliably achieve precise and stable 1-step (20 minutes ahead), 3-step (60 minutes ahead), and 6-step (120 minutes ahead) point and interval forecasting of ST. The architectural diagram is depicted in **Figure 1**.

### 2.2 Study area and data sources

**Figure 2:**
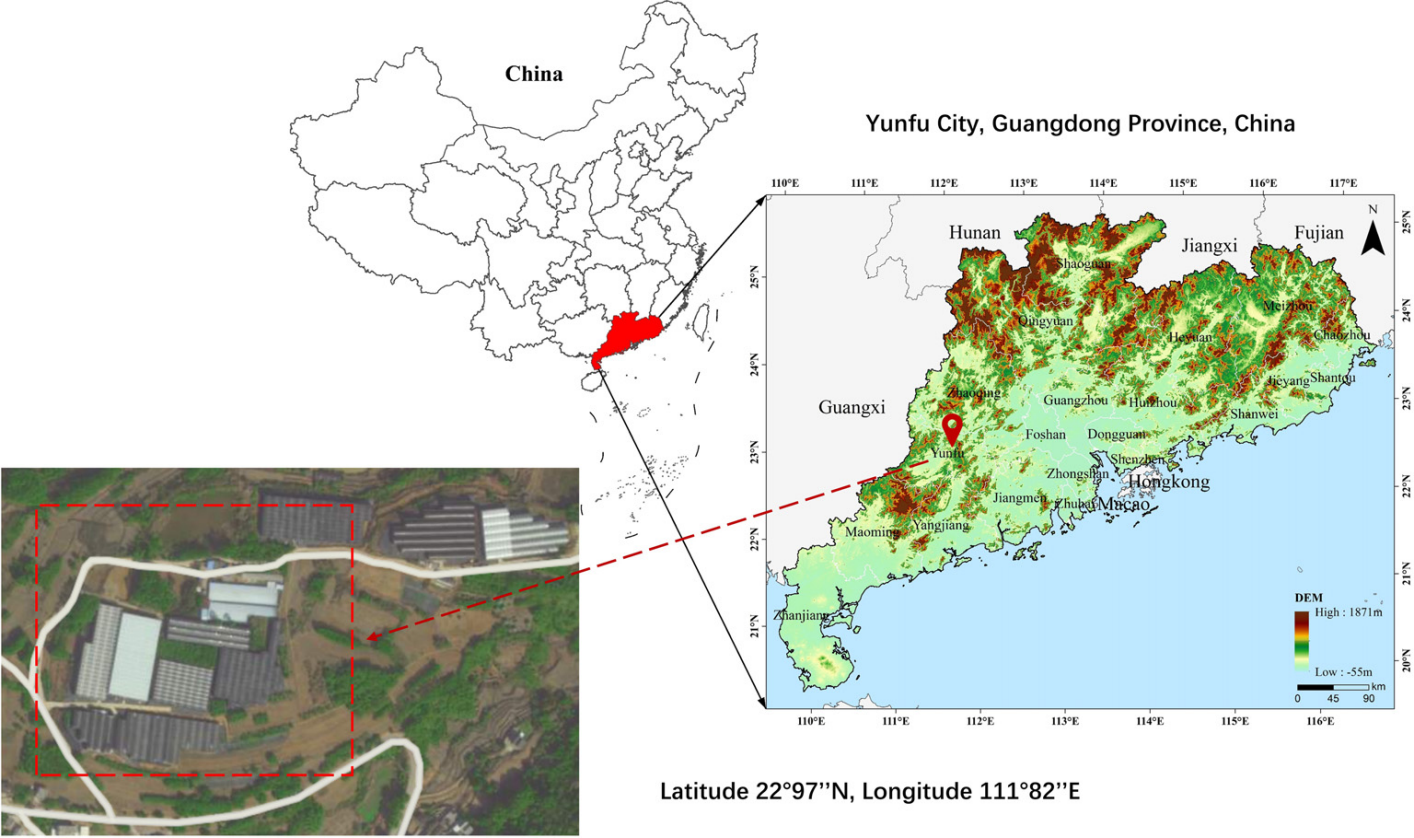
Research area distribution.

The experimental site for this study is located at the plant cultivation base in Yunfu City, Guangdong Province, China (latitude 22°97’’N, longitude 111°82’’E). The distribution and real conditions of the base are illustrated in **Figure 2**. The Big Apple is currently one of the most popular varieties of Aglaonema in the potted plant market (Hui et al., 2023). With its vivid crimson foliage, the Big Apple symbolizes prosperity and good fortune. Placing the Big Apple within homes or gardens not only offers a heightened aesthetic appeal but also carries profound symbolic significance. Thriving in warmth, the Big Apple exhibits remarkable sensitivity to environmental fluctuations. Optimal soil temperatures for its growth range around 27°C during the day and 22°C at night. While it can withstand temperatures as low as 15°C in winter, ensuring a minimum temperature above 20 °C is essential for sustaining its normal growth (You et al., 2024).

**Figure 3:**
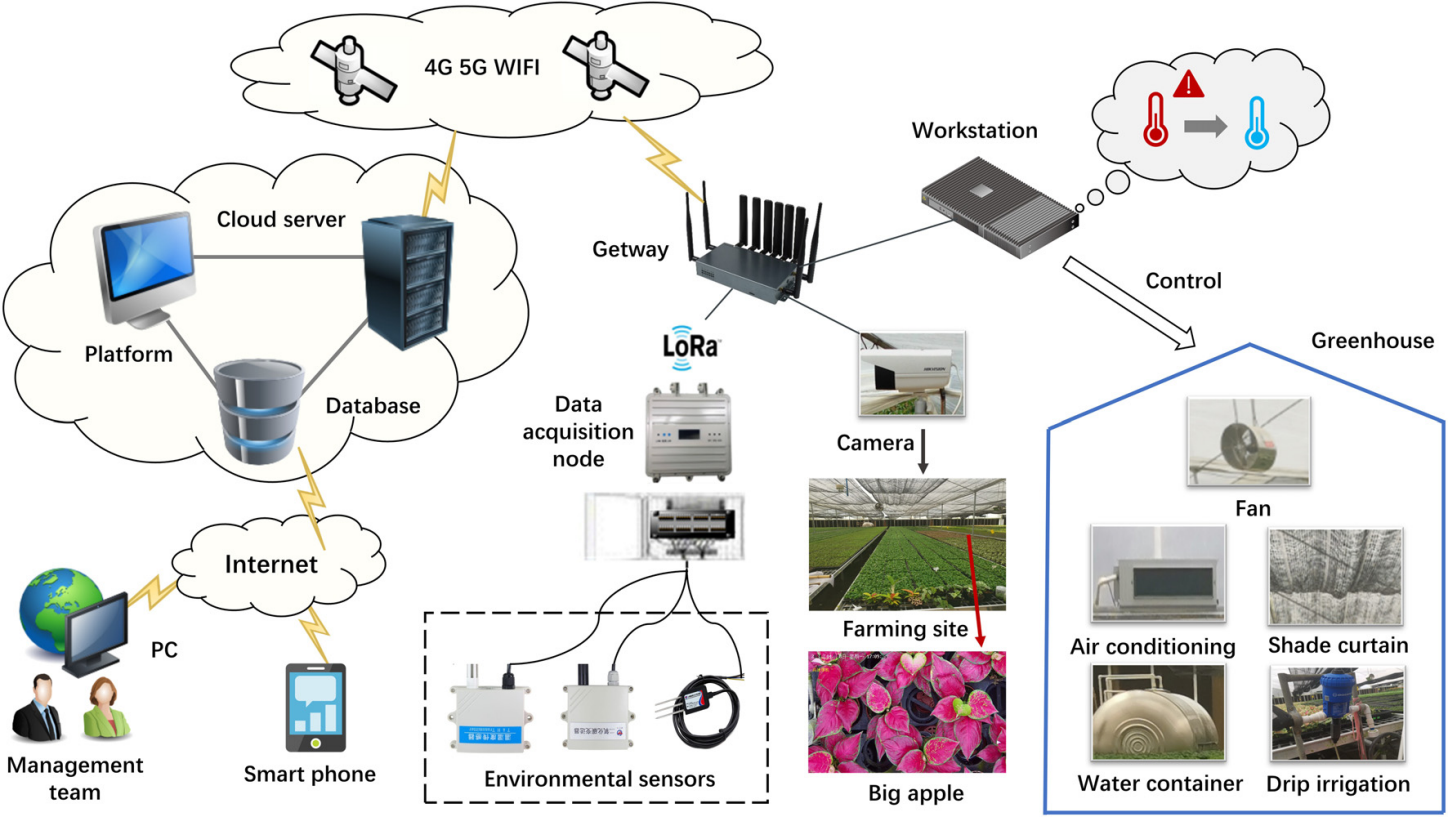
Topology diagram of remote monitoring platform.

To ensure the smooth progression of experiments, research team established a IoT remote monitoring platform. As illustrated in **Figure 3**, we utilized an array of IoT sensors to collect environmental data including air temperature, air humidity, carbon dioxide, soil temperature, soil moisture, and soil conductivity. The specific parameters of the sensor devices are detailed in **Table 1**. Soil monitoring sensors are positioned approximately 15 centimeters deep within the soil, while environmental monitoring sensors are installed at a height of 2.4 meters above the ground, obtaining data with minimal interference (Placidi et al., 2021; Yang et al., 2023). The collected real-time environmental data is transmitted through router nodes to both the on-site monitoring workstation and the remote cloud platform. The on-site monitoring workstation is equipped with relevant predictive algorithms, which facilitate real-time forecasting of environmental factors. It integrates personalized managements with facility equipment (such as water containers, air conditioners, and fans) to achieve effective environmental control and adjustment, thereby precisely establishing the optimal cultivation environment for foliage plants. Moreover, the remote cloud platform enables management personnel to conveniently access and download relevant data via personal computers or mobile devices, facilitating further data analysis and research.

**Table 1:** Internet of things sensor parameters.

Environmental factors	Measurement range	Monitoring accuracy	Protocol
Air humidity (%)	0~100	± 5	IIC
Air temperature (°C)	-40~105	± 0.4	IIC
Carbon dioxide (ppm)	0~50000	± 10	PWM
Soil temperature (°C)	-20~75	± 0.3	Modbus
Soil moisture (%)	0~100	± 5	Modbus
Soil conductivity (µs/cm)	0~999.9	± 5	Modbus

### 2.3 Selection of input variables

Environmental regulation under complex settings in facility cultivation is a typical multivariable time series forecasting task. Combining multiple environmental factors from on-site for multivariate ST forecasting can improve predictive performance. Nevertheless, directly using all environmental variables for training increases both model training time and complexity. Moreover, irrelevant environmental factors could potentially decrease predictive accuracy. Therefore, precise feature selection is crucial. Previous studies predominantly relied on traditional statistical feature selection methods, such as association and correlation analysis (He et al., 2022; Li et al., 2024). Although these methods are simple and convenient, they have several limitations, including stringent statistical assumptions, an emphasis on relationship strength while overlooking trends, and subjectivity in assessing feature importance (Asamoah, 2014). These issues considerably diminish their effectiveness in the complex environments of facility cultivation. Gradient boosting tree models, such as XGBoost (Chen and Guestrin, 2016), LightGBM (Ke et al., 2017), and CatBoost (Prokhorenkova et al., 2018), use decision trees as weak learners. These models not only provide feature importance ranking but, similar to deep learning models, learn by optimizing the loss function. This makes them particularly well-suited for feature extraction in high-dimensional and long data scenarios.

The feature selection approach based on gradient boosting tree models is outlined as follows: XGBoost, LightGBM, and CatBoost models employ split gain as a metric for feature measurement and use ensemble learning to rank the importance of features related to the target. They utilize method of progressively eliminating less important features and iteratively training the model for optimization. This means that the three models gradually select features while observing training results and ensuring that model accuracy remains intact until the best forecasting performance is achieved. This method effectively selects important features contributing to the target while discarding irrelevant ones, thereby reducing data dimensionality and enhancing computational efficiency. The results from the three sets of experiments not only complement each other but also serve as mutual references, ensuring the robustness and reliability of the experiments while preventing experimental randomness.

### 2.4 Soil temperature forecasting model

#### 2.4.1 N-HiTS model

BN Oreshkin et al. (Oreshkin et al., 2019) introduced the N-BEATS model in 2019 for time series forecasting tasks, showcasing state-of-the-art performance on the time series forecasting task. The N-BEATS model not only efficiently handles various types of trends, cycles, and seasonality in time series but also addresses multivariate and long-term forecasting problems more effectively. The core principle of the N-BEATS model lies in its utilization of stacked fully connected neural network blocks, with each block capable of handling patterns at different time scales. This architecture enables the model to discern both short-term fluctuations and long-term trends in time series data. Specifically, the input of each block is the previous block’s input subtracted by the output of the previous block. In this manner, each layer of the block handles the residuals that previous layers failed to properly fit, serving to decompose and forecast the time series in a layered manner. Additionally, the N-BEATS model incorporates multiple forecasting stacks, each tasked with forecasting different features or time ranges within the time series. By employing a weighted loss function to balance the contributions of various scales, the model achieves more precise forecasting.

**Figure 4:**
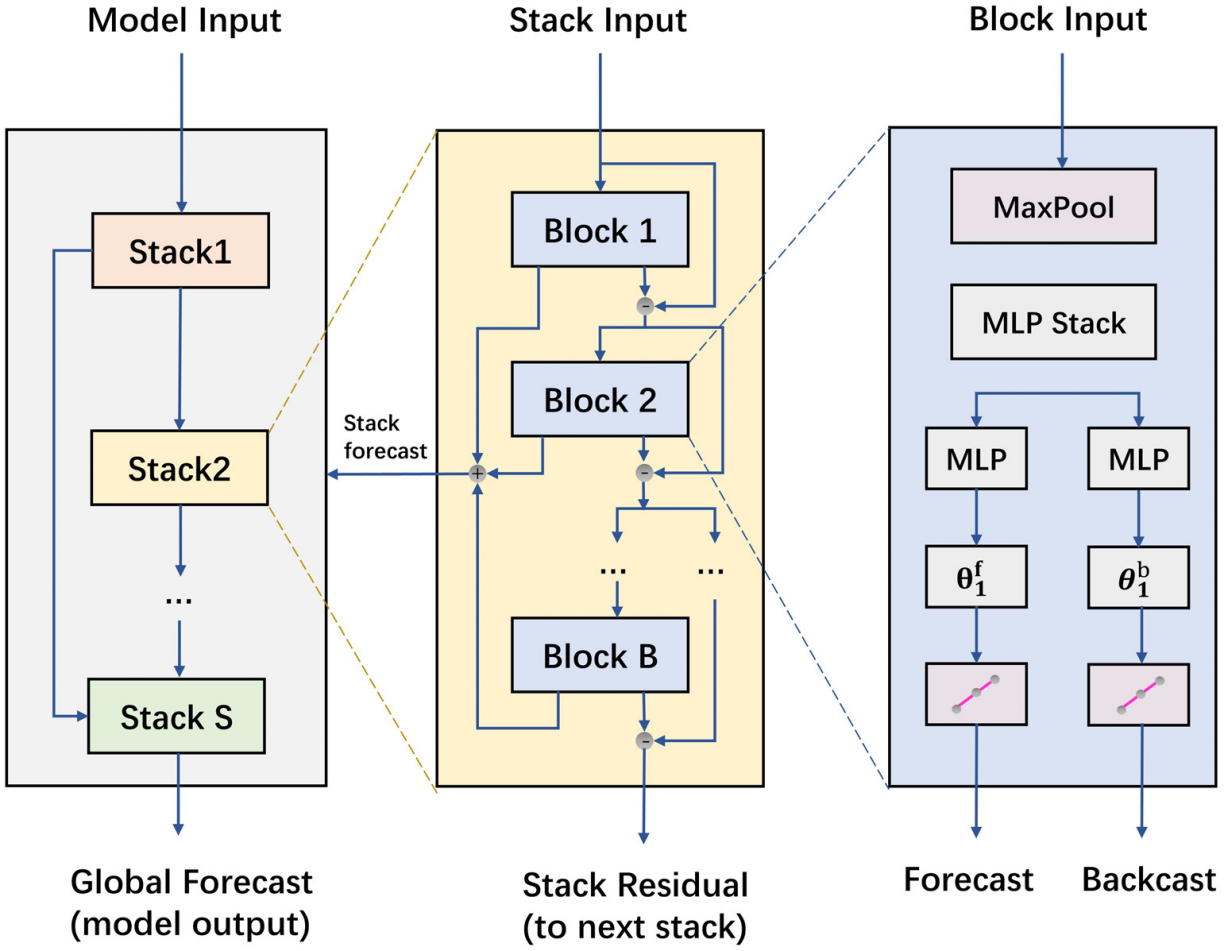
N-HiTS structure.

N-HiTS represents a refinement of the N-BEATS model, aimed at further elevating its multi-step forecasting abilities (Challu et al., 2023). N-HiTS introduces innovative techniques such as multiple-rate sampling and multi-level hierarchical interpolation, which not only reduce computational demands but also enhance predictive accuracy effectively. As shown in **Figure 4**. Specifically, expanding upon the foundation laid by N-BEATS, N-HiTS incorporates a MaxPool layer before each block for pooling operations, effectively sampling the time series into sequences of multiple granularities. The frequency or scale of the time series is related to the pooling kernel size of the MaxPool layer. This approach not only simplifies model training complexity but also boosts forecasting efficiency while curbing the risk of overfitting. Since each input sequence undergoes down-sampling, interpolation is required for the block outputs to up-sample the output quantity to match the forecasting horizon. Overall, the model is capable of efficiently and accurately handling multivariate forecasting tasks, making it well-suited for timely early warning and control in facility cultivation environments.

#### 2.4.2 N-HiTS-Gaussian likelihood distribution model

The N-HiTS model demonstrates swift inference speed and excellent multi-step forecasting capabilities, particularly in capturing temporal trends. However, errors remain inevitable in multi-step forecasting, introducing uncertainty into the assessment and management of soil conditions. Therefore, adopting interval forecasting methods becomes essential to quantify the risks associated with point forecasting errors. In pursuit of soil temperature interval forecasting, we propose an interval forecasting model based on N-HiTS and Gaussian likelihood distribution. The Gaussian likelihood distribution is a novel approach for constructing forecasting intervals, primarily utilizing the Gaussian likelihood function as the loss function to guide the model training and output the probability distribution parameters of future data. The method is characterized by its simplicity in objectives, fewer parameters, and direct output of interval distributions, and high robustness. For clarity in subsequent discussions, we name this model N-HiTS-G, where G represents the Gaussian likelihood distribution.

**Figure 5:**
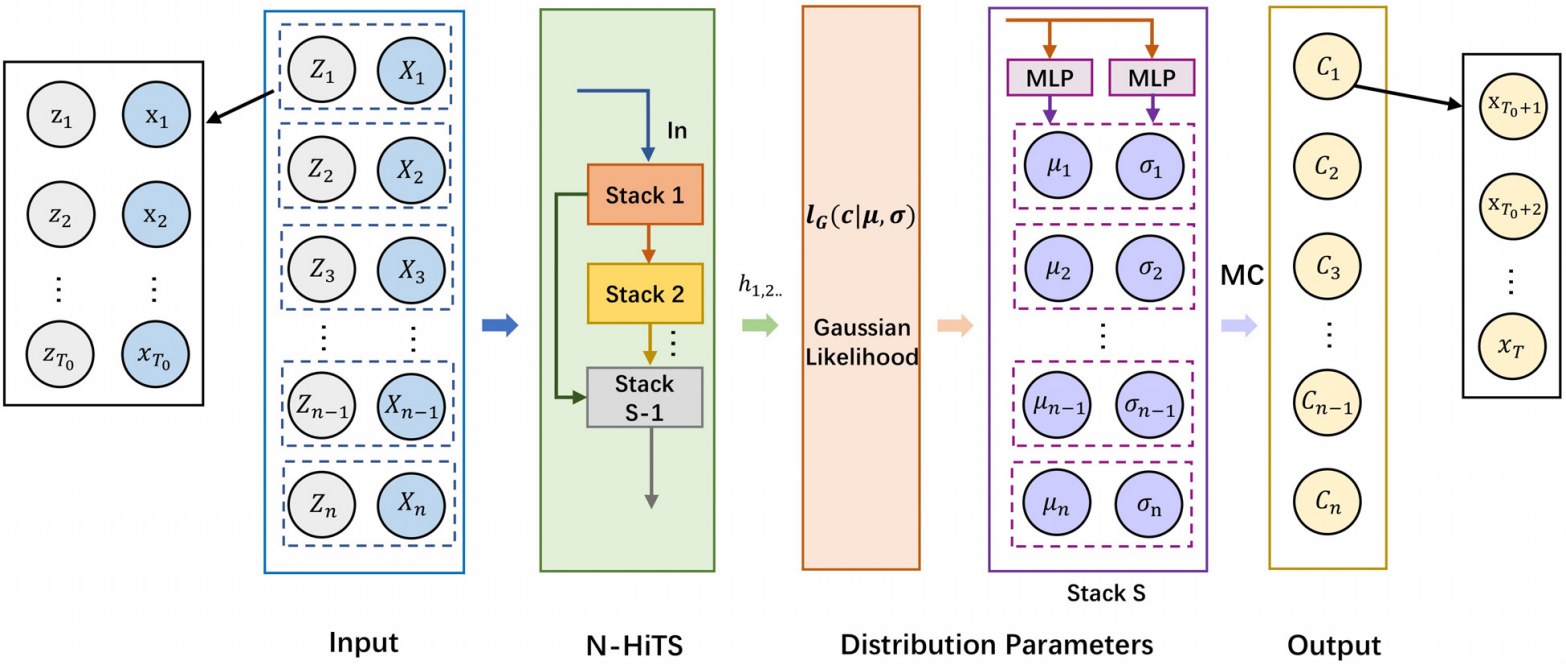
N-HiTS-G structure.


As shown in **Figure 5**, the inputs for training our model primarily consist of historical targets ${\text{X}}_{\text{t}}=\left[{\text{x}}_{1},{\text{x}}_{2},\ldots ,{\text{x}}_{\text{T}0-2},{\text{x}}_{\text{T}0}\right]$ and historical covariates ${\text{Z}}_{\text{t}}=\left[{\text{z}}_{1},{\text{z}}_{2},\ldots ,{\text{z}}_{\text{T}0-2},{\text{z}}_{\text{T}0}\right]$ . ${\text{X}}_{\text{t}}$ and ${\text{Z}}_{\text{t}}$ together form a multivariate time series. Besides, our target forecasting is denoted by ${\text{ C}}_{\text{t}}=\left[{\text{x}}_{{\text{T}}_{0}+1},{\text{x}}_{{\text{T}}_{0}+2},\ldots ,{\text{x}}_{\text{T}-1},{\text{x}}_{\text{T}}\right]$ , representing future data of observations. The primary objective of the model is to forecast the probability distribution p of each subsequent observation for $\text{T}-{\text{T}}_{0}$ based on a historical data sequence of length ${\text{T}}_{0}$ . We can define the probability distribution p for future observations as follows:

(1) $$\begin{array}{c} p_{\Theta }\left(c_{T_{0}:T}|x_{1:T},z_{1:T}\right)=\prod_{t=t0}^{T}p_{\Theta }\left(c_{t}x_{1:t-1},z_{1:t-1}\right)\\ =\prod_{t=t0}^{T}\text{ℓ}(c_{t}|\theta \left(h_{t},\Theta )\right) \end{array}$$

(2) $$h_{t}=h\left(h_{t-1},z_{t-1}x_{t-1},\Theta \right)$$

Here, h represents the N-HiTS forecasting model, ${\text{h}}_{\text{t}}$ signifies the output of N-HiTS at time point t, Θ stands for the model parameters of N-HiTS-G, and θ denotes the likelihood parameters of the Gaussian likelihood function.

Specifically, during training, at each time point, the network input comprising the target data ${\text{X}}_{\text{t}}$ and covariates ${\text{Z}}_{\text{t}}$ from the previous ${\text{T}}_{0}$ time periods, along with the output ${\text{h}}_{\text{t}-1}$ of the neural network at the previous time step. The model’s internal parameters are updated by maximizing the log-likelihood function $l_{G}\left(\text{c|}\mu ,\text{σ}\right)$ . Subsequently, the final layer of the stack is replaced with two multilayer perceptron (MLP) layers, which output the median and variance of the probability distribution for the target values. Monte Carlo (MC) methods are employed to resample the data, generating interval distributions for future target values. During the training of N-HiTS-G, the median of the forecasting interval is used as the point forecast output for fitting the residuals between different blocks. The Gaussian likelihood function $l_{G}\left(\text{c|}\mu ,\text{σ}\right)$ is defined as follows:


(3) $$l_{G}\left(c|\mu ,\sigma \right)=\frac{1}{\sqrt{2\pi \sigma^{2}}}e^{\frac{-{\left(z-\mu \right)}^{2}}{2\sigma^{2}}}$$

As for the parameters of the mean $\text{μ}\left({\text{h}}_{\text{t}}\right)$ and the standard deviation $\text{σ}\left({\text{h}}_{\text{t}}\right)$ in these likelihood functions, we map them through the output ${\text{h}}_{\text{t}}$ of N-HiTS, with the calculation formula as follows:


(4) $$\mu \left(h_{t}\right)=wh_{t}+b$$

(5) $$\sigma \left(h_{t}\right)=lg\left(1+\text{exp}\left(wh_{t}+b\right)\right)$$

Based on this, data analysis of the forecasting interval distribution is conducted. The median of the forecasting interval distribution serves as the point forecasting value. Additionally, a specified confidence level is set to derive the final forecasting interval. This confidence level not only provides the range where the target is likely to lie but also indicates its accuracy, offering richer and more reliable information than point forecasts, which aids in risk management.

#### 2.4.3 Soil temperature forecasting based on SP-N-HiTS-G model

##### 2.4.3.1 Parameter determination issue

Although N-HiTS-G has the potential to demonstrate superior point forecasting and interval forecasting performance, it is constrained by initialization parameters. Due to the continual stacking of MLP blocks and utilization of pooling layers, N-HiTS-G involves a greater number of hyperparameters compared to typical time series forecasting models. The increased scale and sensitivity of the hyperparameters significantly reduces the practicality of traditional grid search methods. Therefore, to address the issue of parameter determination, this study employs the multi-objective optimization algorithm to optimize the initialization parameters of N-HiTS-G. In order to simultaneously ensure both the point and interval forecasting performance of N-HiTS-G, the optimization objectives of optimization algorithm are defined as the point forecasting metric MAE and the interval forecasting metric CWC.

##### 2.4.3.2 Speed-constrained multi-objective particle swarm optimization algorithm

Particle Swarm Optimization (PSO) is a swarm intelligence optimization algorithm inspired by the foraging behavior of birds (Kennedy and Eberhart, 1995). This algorithm mimics the behavior of birds searching for food in a search space. When the bird swarm does is unaware of the exact location of food, individual birds rely on their memory of the best position and the collective experience of the bird swarm to search for food.

To further expand the application of the PSO algorithm, scholars have endeavored its applicability to Multi-objective optimization. They have proposed the Multi-objective Particle Swarm Optimization (MOPSO) algorithm, incorporating the notions of external archives and the Pareto dominance principle (Coello and Lechuga, 2002). Despite its merits, MOPSO encounters challenges related to convergence and search capabilities. In response, Myster and Moe (1995) introduced the Speed-constrained Multi-objective Particle Swarm Optimization (SMPSO) as an enhancement to MOPSO. Building upon the foundation of MOPSO, SMPSO imposes constraints on particle velocities to prevent them from straying beyond the feasible solution space, thereby ensuring the stability of particle movement.

##### 2.4.3.3 Operational steps of the SP-N-HiTS-G model

**Figure 6:**
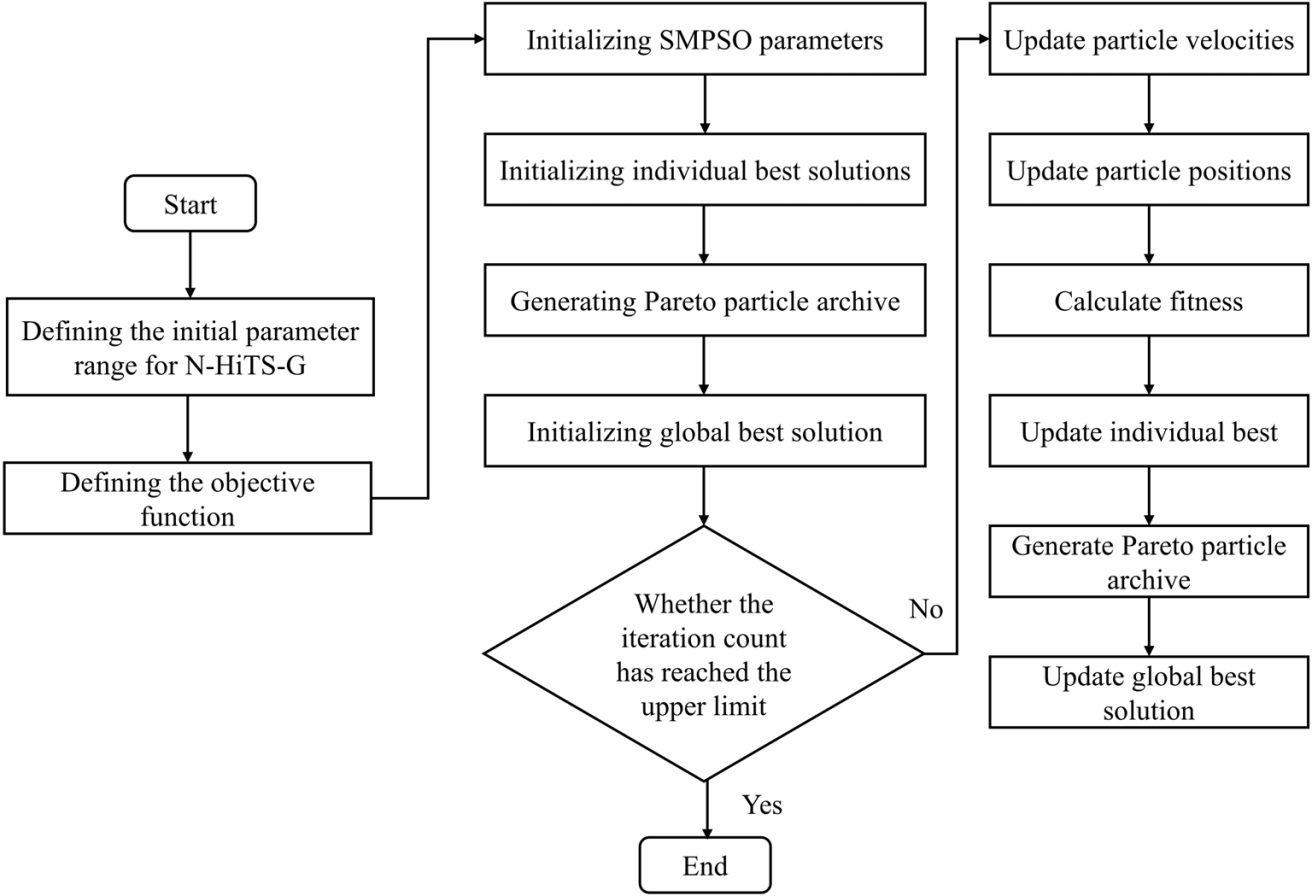
The flowchart of the SP-N-HiTS-G model.

To search for the optimal hyperparameters of N-HiTS-G, start by defining the hyperparameter space as a unified entity, where each instance represents a unique combination of hyperparameters. Next, utilize the SMPSO algorithm to initialize this space, evaluating the fitness of each hyperparameter combination. Subsequently, refine both the particle swarm information and the Pareto archive. For ease of discussion, we refer to the N-HiTS-G model optimized by SMPSO as the SP-N-HiTS-G model, where SP denotes the SMPSO algorithm. The optimization process of SP-N-HiTS is shown in **Figure 6**, with the optimization approach outlined as follows:

Step 1: Define the hyperparameters of the N-HiTS-G model to be optimized along with their ranges, setting Mean Absolute Error (MAE) and Coverage Width Standard (CWC) as objective functions. The parameter search range for N-HiTS-G is outlined in **Table 2**, with all parameters ultimately yielding integer values.

**Table 2:** Search range of hyperparameters for N-HiTS-G.

hyperparameters	Describe	Range
n_blocks	Number of blocks for each stack	[1-5]
mlp_units	Structure of hidden layers for each stack type	[100-500]
n_pool_kernel_size	List with the size of the windows to take a max over	[2-5]
n_freq_downsample	List with the stack’s coefficients	[1-5]
lookback period	Time window of historical data used to predict future data	[5-24]

Step 2: Initialize the parameters of SMPSO, update the individual best positions and global best position of the particle swarm, and establish the Pareto particle archive.

Step 3: Iteratively update the individual information (velocity, position) of the particle swarm and the global best position.

Step 4: Calculate MAE and CWC of N-HiTS-G based on the parameter combinations optimized by SMPSO.

Step 5: Determine if the maximum iteration count has been reached. If satisfied, proceed to Step 6; otherwise, return to Step 3 for further iteration.

Step 6: Conclusion, returning the optimal parameter combination for the N-HiTS-G model.

### 2.5 Data preprocessing

#### 2.5.1 Missing data repair

The experiment utilized greenhouse environmental data collected from February 14, 2024, to May 4, 2024, with a sampling interval of 20 minutes, yielding a total of 5767 data points. Due to the influence of climate variations and network fluctuations, a negligible amount of data was unavoidably lost during the data collection process by the IoT sensors. However, leaving these gaps untreated would render the model unable to train. Based on the inherent characteristics of time series data, this study employed linear interpolation to fill in missing values. Assuming there is a missing value y at a certain position x between coordinates $\left({\text{x}}_{0},{\text{ y}}_{0}\right)$ and $\left({\text{x}}_{1},{\text{y}}_{1}\right)$ , the linear interpolation formula is:


(6) $$y=y_{0}+\left(x-x_{0}\right)\frac{y_{1}-y_{0}}{x_{1}-x_{0}}$$

#### 2.5.2 Data and normalization

This study selected the first 4036 data samples (70%) as the training set and 866 data samples (15%) as the validation set for model parameter tuning. Subsequently, 865 data samples (15%) were chosen as the test set to assess and compare the point and interval forecasting performance of various models. To address potential inconsistencies in data dimensions and enhance forecasting model performance, we applied Min-Max normalization for data scaling.


(7) $$x_{new}=\frac{x-x_{min}}{x_{max}-x_{min}}$$

### 2.6 Evaluation metrics

#### 2.6.1 Point forecasting evaluation metrics

In this study, we evaluate the performance of point forecasting models using Root Mean Square Error (RMSE), Mean Absolute Error (MAE), and Mean Absolute Percentage Error (MAPE). The calculation formulas are as follows:


(8) $$\text{RMSE}=\sqrt{\frac{1}{n}\sum_{i=1}^{n}{\left(Y_{i}-y_{i}\right)}^{2}}$$

(9) $$MAE=\frac{1}{n}\sum_{i=1}^{n}|Y_{i}-y_{i}|$$

(10) $$MAPE=\frac{100\%}{n}\sum_{i=1}^{n}\left|\frac{Y_{i}-y_{i}}{y_{i}}\right|$$

Here, ${\text{Y}}_{\text{i}}$ denotes the predicted value at time point i, while ${\text{y}}_{\text{i }}$ represents the observed value at time point i.

#### 2.6.2 Interval forecasting evaluation metrics

This study evaluates the performance of interval forecasting models using Prediction Interval Coverage Probability (PICP), Prediction Interval Normalized Root Width (PINRW), Prediction Interval Normalized Average Width (PINAW), and Coverage Width Standard (CWC). Specifically, PICP reflects the coverage probability of the forecasting interval for observed values, with values ranging from 0 to 1. A higher PICP suggests that more observed values fall within the interval, indicating better forecasting performance. PINAW and PINRW metrics evaluate the width of the forecasting interval. A excessively wide forecasting interval diminishes the credibility of the information and fails to effectively characterize uncertainty. CWC, on the other hand, serves as a comprehensive metric that simultaneously considers both the coverage probability and width of the forecasting interval, providing a more intuitive measure of the forecasting interval quality.


(11) $$\begin{array}{l} \text{PICP}=\frac{1}{\text{N}}\left(\sum_{i=1}^{N}{\text{C}}_{\text{i}}\right)\\ {\text{C}}_{\text{i}}=\{\begin{array}{cc} 1, & {\text{y}}_{\text{i}}\in \left[{\text{y}}_{\text{i}}^{\text{U}},{\text{y}}_{\text{i}}^{\text{L}}\right]\\ 0, & {\text{y}}_{\text{i}}\notin \left[{\text{y}}_{\text{i}}^{\text{U}},{\text{y}}_{\text{i}}^{\text{L}}\right] \end{array} \end{array}$$

(12) $$PINAW=\frac{1}{\text{N}\text{A}}\sum_{\text{i}=1}^{\text{N}}\left|{\text{y}}_{\text{i}}^{\text{U}}-{\text{y}}_{\text{i}}^{\text{L}}\right|$$

(13) $$\text{PINRW}=\frac{1}{\text{A}}\sqrt{\frac{1}{N}\sum_{\text{i}=1}^{\text{N}}{\left({\text{y}}_{\text{i}}^{\text{U}}-{\text{y}}_{\text{i}}^{\text{L}}\right)}^{2}}$$

(14) $$\begin{array}{l} \text{CWC}=\text{PINAW}\left(1+\text{γ}{\text{e}}^{-\text{η}\left(\text{PICP}-\text{μ}\right)}\right)\\ \text{γ}=\{\begin{array}{cc} 0, & \text{PICP}\geq \text{μ}\\ 1, & \text{PICP}<\text{μ} \end{array} \end{array}$$

${\text{X}}_{\text{i}}$ represents the true value of the target variable, $y_{i}^{U}$ and $y_{i}^{L}$ represent the upper and lower bounds of the forecasting interval, respectively. A is the range of the target values used for data normalization, μ is the minimum threshold for the PICP, set here as 0.80. A low PICP indicates a lack of confidence in the forecasting interval. η is the penalty coefficient for forecasting intervals with low PICP, set to 1 in this experiment. If the PICP is satisfactory, the CWC is not affected by the PICP.

## 3 Result and discussion

### 3.1 Experimental environment

The experiment was conducted on a workstation running Ubuntu 18.04 Linux operating system, featuring an Intel(R) I7-13700H 5.0 GHz CPU, 16GB RAM, and an NVIDIA GeForce RTX3060 GPU. The algorithmic model was trained and tested in an environment utilizing Python 3.8.5, Scikit-Learn 1.1.1, and PyTorch 2.1.0. During the process of feature selection, experiments were performed using default parameters of XGBoost, LightGBM, and CatBoost.

### 3.2 Analysis and selection of important environmental factors

**Figure 7:**
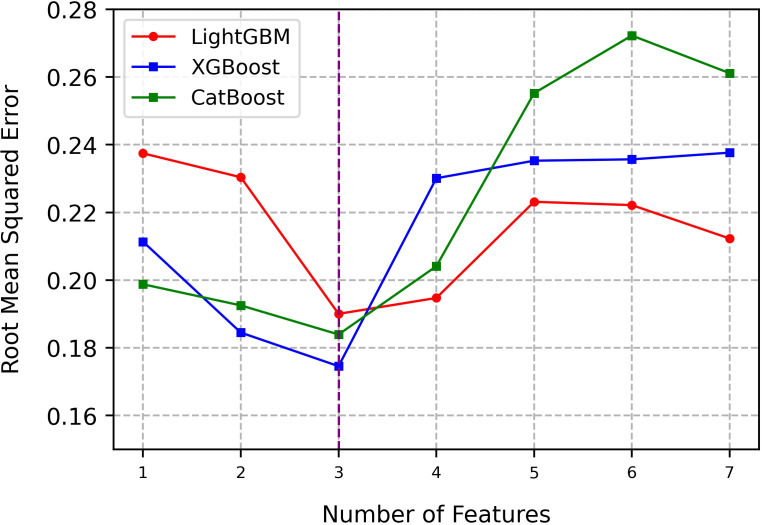
Comparison of performance for different feature combinations.

ST forecasting can be regarded as a time series forecasting problem, utilizing environmental data from previous time periods, including ST and other environmental factors, to forecast ST for the subsequent time period. Therefore, the initial step involves transforming the raw data into supervised learning data using a time lag method, followed by training with XGBoost, LightGBM and CatBoost models. During the training process, we obtained rankings for feature importance and iteratively reduced the number of features. The RMSE was used as the evaluation metric to assess the predictive performance of the model. The experimental results are presented in **Table 3** and **Figure 7**. Although there are slight differences in the feature importance scores among the three models, they exhibit a consistent overall ranking of feature importance. From highest to lowest importance, the order of features is consistently as follows across all models: soil temperature, air temperature, air humidity, soil moisture, light, soil conductivity, and carbon dioxide.

**Table 3:** Ranking of the importance of different environmental factors.

Environmental factors (previous time period)	LightGBM	XGBoost	CatBoost
Soil temperature	0.256	0.868	0.670
Air temperature	0.147	0.085	0.229
Air humidity	0.147	0.024	0.029
Soil moisture	0.127	0.015	0.028
Light	0.127	0.004	0.020
Soil conductivity	0.125	0.001	0.124
Carbon dioxide	0.069	3*10-4	0.009

Moreover, from **Figure 7**, we observe that regardless of whether it is XGBoost, LightGBM, or CatBoost, when the number of features is set to three, including the previous soil temperature, previous temperature, and previous humidity, the three models exhibit the best forecasting performance. Their RMSE values are 0.175, 0.183, and 0.192 respectively. In contrast, other feature combinations result in increased forecasting errors. Therefore, in this experiment, utilizing previous soil temperature, previous air temperature, and previous air humidity data as crucial features, and feeding them into the final forecasting model to enhances ST predictive accuracy. We will further demonstrate the efficacy of this feature selection method in Experiment 3.6.

### 3.3 Performance of different forecasting models of soil temperature

To validate the point and interval forecasting performance of the SP-N-HiTS-G model, this study conducted comprehensive experiments and comparisons with a range of common and advanced forecasting models. For point forecasting, the comparative models primarily including ARIMA, LSTM, GRU, LSTM-Attention, N-HiTS, Temporal Fusion Transformer (TFT) (Lim et al., 2021), and Informer (Zhou et al, 2021). ARIMA, LSTM, GRU, and LSTM-Attention are among the most widely used time series models in past studies on greenhouse environment forecasting (Zeynoddin et al., 2020; Liu et al., 2022; He et al., 2022; Yang et al., 2023), while TFT and Informer represent advanced forecasting models known for their innovative use of attention mechanisms. By optimizing the attention mechanism module, their excellent forecasting capability has been validated in various fields, including energy and transportation (Wei et al., 2023; Zhang et al., 2022).

For interval forecasting, the study compared the SP-N-HiTS-G model with DeepAR and MQRNN models (Salinas et al., 2020; Wen et al., 2017). DeepAR, similar to our proposed model, employs maximum likelihood estimation for updating parameters in interval forecasting. However, it utilizes the LSTM as a feature extractor and follows a recursive forecasting approach. In addition, the MQRNN model combines efficient local information handling using CNNs with sequence modeling capabilities of RNNs, demonstrating commendable forecasting performance. Furthermore, the experiments were complemented with three commonly used interval construction methods: error fitting, bootstrap, and quantile Loss method (Niu et al., 2022; Xiao et al., 2022; Li et al., 2023). The error fitting method primarily constructs intervals using parameters or non-parameter fitting results of point forecasting errors, often employing kernel density estimation. Bootstrap, on the other hand, approximates forecasting intervals by resampling and randomly selecting results from point forecasting errors. Moreover, The quantile loss is a regression loss function based on quantiles. It partitions predicted values into different quantiles and measures the loss of actual values at corresponding quantiles in the predicted distribution. For clarity in subsequent discussions, we named these three interval construction method models respectively as N-HiTS-E (E for error fitting), N-HiTS-B (B for bootstrap), and N-HiTS-Q (Q for quantile loss).

**Figure 8:**
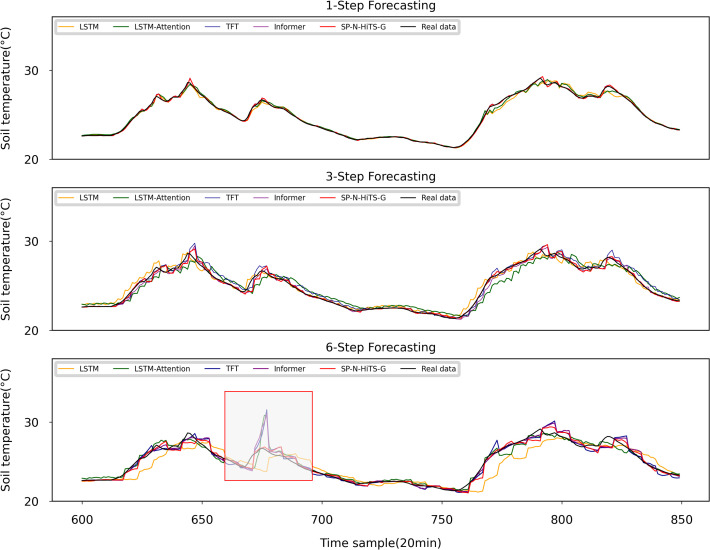
Point forecasting results of different models.

To ensure the fairness and rigor of the experiments, we maintained consistency across all models by using identical inputs and outputs. Furthermore, we used an early stopping mechanism to prevent over-fitting and promote model generalization. If the model performance on the validation set does not improve for more than 50 epochs, training was terminated to reduce unnecessary computational expenses. This study encompassed three distinct forecasting tasks: 1 step (20 minutes ahead) forecasting, 3-step (60 minutes ahead) forecasting, and 6-step (120 minutes ahead) forecasting of ST. Regarding the hyperparameter selection for models other than SP-N-HiTS, a hybrid approach combining grid search and manual fine-tuning methods was adopted. This method enabled us to explore optimal parameters within a reasonable range for the baseline forecasting models. The experimental findings are presented in **Tables 4**–**6**, and **Figure 8**, we observe that all models perform well in single-step forecasting. However, as the forecast horizon extends, the predictive performance of ARIMA deteriorates significantly, revealing the difficulty of mathematical models in achieving accurate multivariate multi-step forecasts. Furthermore, the forecasting curves of models from previous studies, such as LSTM and LSTM-Attention, exhibit a significant decline in their alignment with the original values, accompanied by substantial fluctuations, which indicates instability and reduces their practical value. Overall, the SP-N-HiTS-G model proposed in this study exhibits the most stable point forecasting performance across varying forecast horizons, surpassing both models from prior research and other advanced forecasting approaches.

**Table 4:** Experimental results of 1-step soil forecasting (20 minutes ahead).

Model	RMSE	MAE	MAPE	PICP	PINRW	CWC	Train-time (s)
ARIMA	0.204	0.136	0.504	/	/	/	19.12
LSTM	0.189	0.122	0.488	/	/	/	23.34
GRU	0.191	0.122	0.488	/	/	/	22.45
LSTM-Attention	0.149	0.103	0.415	/	/	/	44.26
TFT	0.086	0.057	0.230	/	/	/	62.32
Informer	**0.085**	**0.056**	**0.229**	/	/	/	168.00
N-HiTS	0.160	0.090	0.361	/	/	/	20.62
DeepAR	0.092	0.064	0.262	0.812	0.053	0.031	32.97
QRNN	0.089	0.058	0.241	0.812	0.053	0.031	33.88
N-HiTS-E	/	/	/	0.814	0.041	0.027	20.73
N-HiTS-B	/	/	/	0.812	0.043	0.028	20.81
N-HiTS-Q	0.092	0.064	0.264	0.812	0.037	0.025	20.92
N-HiTS-G	0.089	0.061	0.252	0.813	0.033	0.023	22.43
**SP-N-HiTS-G**	0.087	0.057	0.231	0.823	0.033	**0.019**	**17.12**

The bold represent superior performance.

**Table 5:** Experimental results of 3-step soil forecasting (60 minutes ahead).

Model	RMSE	MAE	MAPE	PICP	PINRW	CWC	Train-time (s)
ARIMA	0.478	0.401	1.789	/	/	/	20.34
LSTM	0.409	0.274	1.107	/	/	/	24.35
GRU	0.363	0.277	1.150	/	/	/	23.78
LSTM-Attention	0.339	0.272	1.132	/	/	/	45.32
TFT	0.323	0.219	0.869	/	/	/	67.68
Informer	0.243	0.150	0.606	/	/	/	182.59
N-HiTS	0.247	0.151	0.608	/	/	/	23.91
DeepAR	0.291	0.180	0.720	0.700	0.069	0.105	32.28
QRNN	0.292	0.190	0.764	0.754	0.075	0.112	37.83
N-HiTS-E	/	/	/	0.784	0.087	0.069	24.02
N-HiTS-B	/	/	/	0.783	0.087	0.070	24.08
N-HiTS-Q	0.277	0.157	0.629	0.737	0.069	0.103	22.85
N-HiTS-G	0.241	0.137	**0.541**	0.853	0.082	0.057	20.99
**SP-N-HiTS-G**	**0.237**	**0.136**	**0.541**	0.815	0.077	**0.053**	**18.95**

The bold represent superior performance.

**Table 6:** Experimental results of 6-step soil forecasting (120 minutes ahead).

Model	RMSE	MAE	MAPE	PICP	PINRW	CWC	Train-time (s)
ARIMA	0.622	0.503	2.176	/	/	/	22.67
LSTM	0.479	0.379	1.581	/	/	/	28.38
GRU	0.539	0.464	1.938	/	/	/	29.30
LSTM-Attention	0.468	0.316	1.295	/	/	/	51.38
TFT	0.461	0.270	1.077	/	/	/	72.35
Informer	0.510	0.325	1.271	/	/	/	180.58
N-HiTS	0.445	0.270	1.076	/	/	/	33.76
DeepAR	0.562	0.357	1.422	0.724	0.141	0.228	28.97
QRNN	0.538	0.272	1.068	0.751	0.105	0.153	38.71
N-HiTS-E	/	/	/	0.782	0.105	0.121	24.36
N-HiTS-B	/	/	/	0.786	0.108	0.120	24.86
N-HiTS-Q	0.497	0.285	1.146	0.775	0.113	0.163	25.71
N-HiTS-G	0.420	0.254	1.016	0.802	0.115	**0.077**	22.17
**SP-N-HiTS-G**	**0.393**	**0.243**	**0.973**	0.801	0.116	**0.077**	**21.72**

The bold represent superior performance.

### 3.4 Analysis of point forecasting model performance for different models

In single step forecasting, as illustrated in **Table 4**, the SP-N-HiTS-G model demonstrates excellent point forecasting performance to the advanced attention models such as TFT and Informer. Compared to the widely used LSTM model in previous studies, the SP-N-HiTS-G model demonstrates significant improvements in RMSE, MAE, and MAPE, with reductions of 0.102, 0.065, and 0.257, respectively. These results highlight the effectiveness of the method for ultra-short-term forecasting tasks. Furthermore, in contrast to the Informer model, which boasts optimal short-term point forecasting performance, the SP-N-HiTS-G model exhibits a significant reduction in training time on the workstation, decreasing from 168.0 s to 17.18 s. This acceleration in training speed caters to the need for swift decision-making and precise regulation in facility cultivation environments.

In the 3-step forecasting task, our proposed model exhibits optimal forecasting performance, with RMSE, MAE, and MAPE values of 0.237, 0.136, and 0.541, respectively. Compared to the LSTM model, the SP-N-HiTS-G model also shows significant improvements in RMSE, MAE, and MAPE, with reductions of 0.198, 0.195, and 0.815, respectively, indicating a significant enhancement in short-term multi-step predictive accuracy. Besides, the Informer model was the traditionally top-performing model for 3-step forecasting tasks, but the SP-N-HiTS-G model surpasses it. Specifically, in comparison to the Informer model, The SP-N-HiTS-G model exhibits increases of 2.4% in RMSE, 9.3% in MAE, and 10.7% in MAPE when forecasting the next 3-step. Moreover, the training time has been substantially reduced from 182.59s to 18.95s, representing a minor improvement in predictive accuracy alongside a significant reduction in training time.

In terms of 6-step forecasting, the performance of the SP-N-HiTS-G model significantly outpaces that of other baseline models. Its predicted curve closely tracks the trend of the actual curve, showcasing characteristics of high precision, stability, and rapid inference. Compared to LSTM model, the SP-N-HiTS-G model demonstrates reductions of 0.086, 0.133, and 0.608 in RMSE, MAE, and MAPE, respectively, for forecasting the next 6-step. Following closely behind the N-HiTS series models, TFT model emerges as the second most accurate model for 6-step forecasting. Compared to TFT model, the SP-N-HiTS-G model exhibits a 14.7%, 10%, and 9.6% increase in RMSE, MAE, and MAPE for forecasting the next 6 steps, respectively, while the training time decreases from 72.35 s to 21.72 s. In summary, the SP-N-HiTS-G model proposed in this study exhibits excellent and stable point forecasting performance across various time scales. Besides, compared to all attention mechanism models, the SP-N-HiTS-G model offers faster training speed, thus effectively meeting the demands for model parameter updates and real-time inference based on new data. This approach enables real-time training and updating of the model during data collection intervals, which holds significant value for more precise soil management practices.

**Table 6** and **Figure 8** illuminate on a notable trend: common benchmark models like LSTM and GRU exhibit a significant increase in errors, particularly in MAE and MAPE metrics, when they are used for 6-step forecasting. This suggests that these benchmark models struggle in capturing the cyclical patterns inherent in lengthy time series, leading to significant deviations (Javed et al., 2022). Moreover, **Figure 8** highlights a distinct behavior observed in models incorporating attention mechanisms, including LSTM-Attention, Informer and TFT. They tend to manifest errors at the sharp edges of the test data, resulting in higher RMSE metrics. This inclination towards over-fitting arises from the attention mechanisms’ tendency to excessively exploit correlations between environmental factors and soil temperature. Such over-fitting is elusive during training, despite efforts to mitigate it using validation sets (Liu et al., 2024). In contrast, the N-HiTS model adopts a unique approach to feature extraction, steering clear of an overemphasis on deep feature extraction. Instead, it focuses on extracting temporal features from multiple time scales and employs pooling layers to mitigate excessive data information. This strategy yields greater stability in medium to long -term forecasting performance. Although it may lag slightly in single-step forecasting tasks, N-HiTS consistently demonstrates robustness and reliability in multi-step forecasting tasks.

Furthermore, **Table 6** also reveals an interesting phenomenon worth noting. In contrast to its performance in 1-step to 3-step forecasting, DeepAR exhibits a notable decline in accuracy when tasked with 6-step forecasting. The decline can be attributed to DeepAR utilizing a recursive forecasting method, where the model recursively incorporates uses predicted values to forecast the next predicted value (In and Jung, 2022). The flaw in this approach lies in its heavy reliance on the feature extraction performance of the underlying model. Moreover, for data such as soil temperature which is nonlinear and highly volatile, the errors gradually accumulate as the forecasting steps increase, making it challenging to achieve accurate multi-step forecasting tasks of soil temperature. In contrast, the proposed model adopts a direct forecasting method, utilizing past data to directly forecast all future observations within the forecast horizon. Although this method is simple, it has proven to be effective, contributing to the optimization and reduction of errors during model training (Yaghoubirad et al., 2023). While its advantages may not be as apparent in 1-step to 3-step forecasting tasks, they become evident in the form of enhanced robustness and stability in 6-step forecasting endeavors.

### 3.5 Analysis of interval forecasting model performance for different models

Observations from **Figure 8** reveal the adeptness of various benchmark models in handling 1-step forecasting tasks. However, as the forecasting horizon extends, discernible variances emerge in the forecasting efficacy among these benchmarks. Moreover, all models invariably generate some degree of point forecasting errors in multi-step forecasting tasks, particularly within regions of significant ST fluctuations. Therefore, the necessity of utilizing interval forecasting methodologies becomes more apparent in multi-step forecasting endeavors, facilitating a quantification of risks associated with multi-step forecasting inaccuracies.

**Figure 9:**
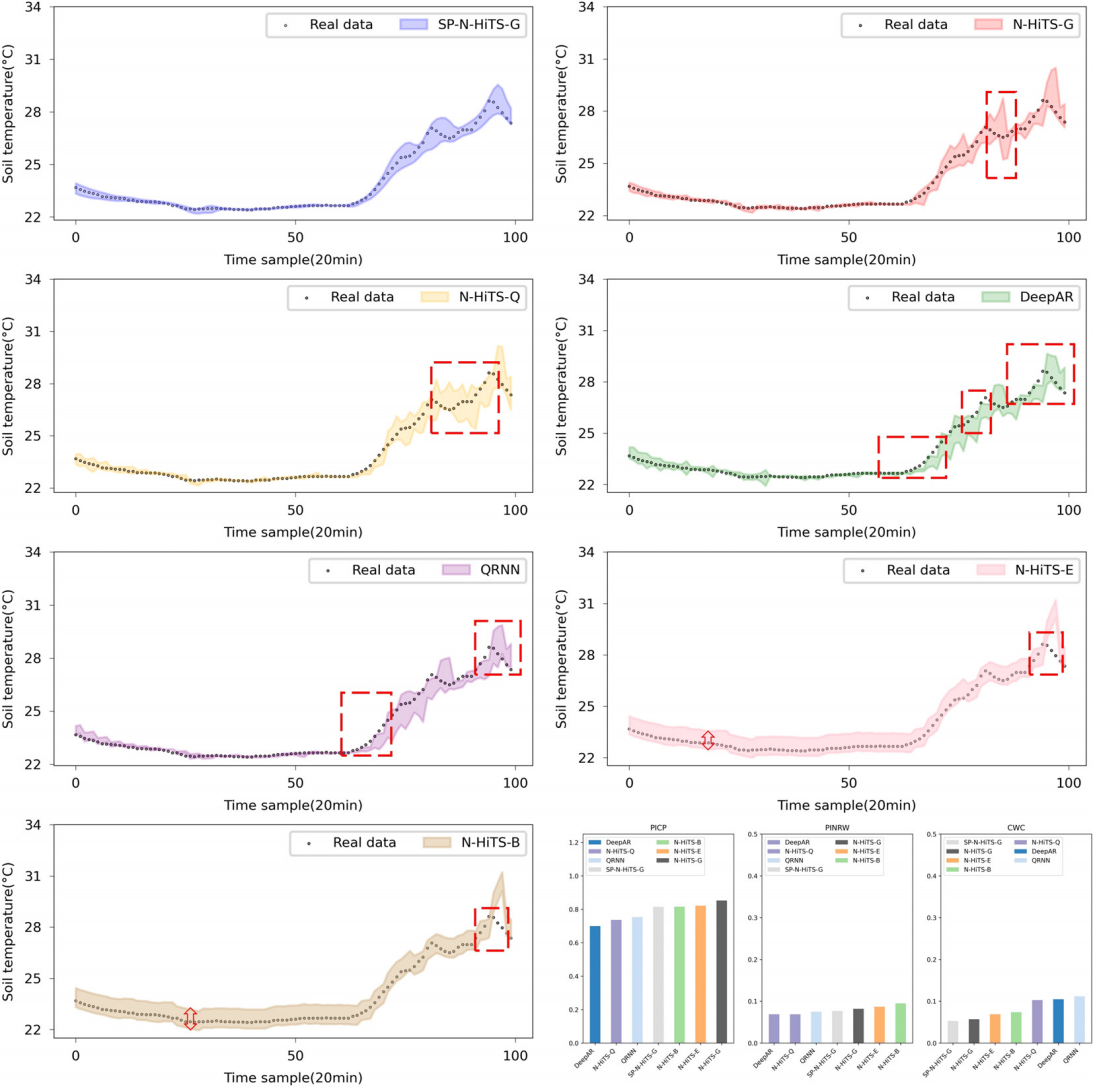
Performance comparison of different Interval forecasting methods in 3-step forecasting.

**Figure 10:**
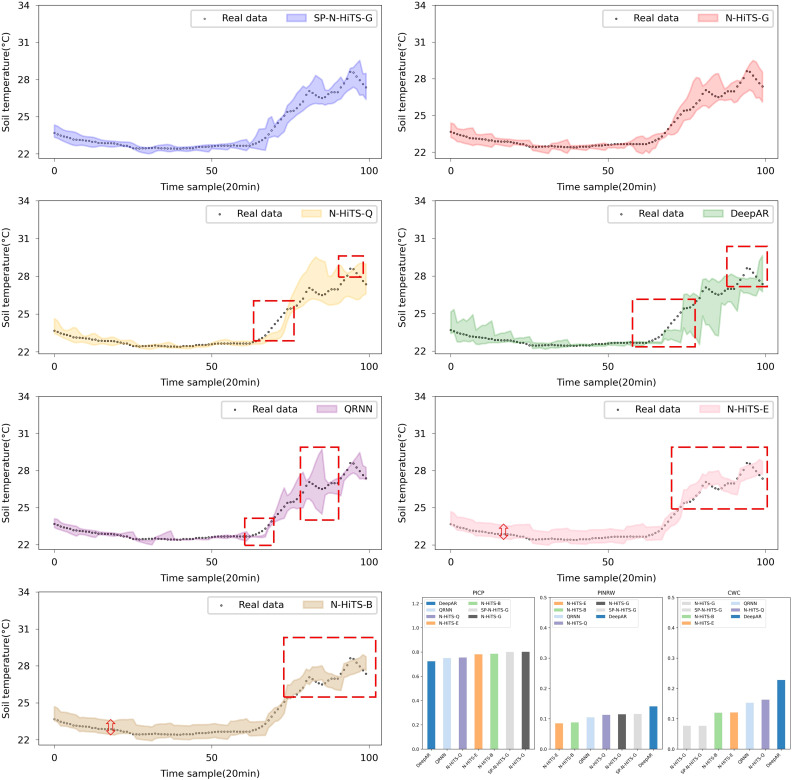
Performance comparison of different Interval forecasting methods in 6-step forecasting.

In regard to interval forecasting, it is evident from **Tables 5** and **6** that, owing to the irregular variations in the ST data, especially its instability at the peaks and corners of the change curve, common interval forecasting methods typically demonstrate a low PICP, particularly noticeable in multi-step forecasting scenarios. **Figures 9** and **10** illustrate the multi-step interval forecasting effects of different methods. It is worth noting that, with a 90% confidence level, the DeepAR model, QRNN model, and N-HiTS-Q model consistently show a low PICP, whereas the PINRW and CWC metrics are excessively high. This indicates that the forecasting intervals generated by these three models neither sufficiently encompass the observed values nor are they of appropriate width, resulting in a lack of credibility and stability. Therefore, they fail to accurately quantify the bias introduced by point forecasting. Although the forecasting intervals formed based on Error Fitting and Bootstrap exhibit an overall good width, they display locally excessive intervals within the stable forecasting range. This occurs because, during the experiment, the validation set error does not perfectly mirror that of the test set. The fixed addition and subtraction of error values amplify the uncertainty within the forecasting intervals, lacking the requisite flexibility and diversity.

The forecasting interval generated by the SP-N-HiTS-G model adeptly balances high interval coverage rates with appropriately narrow interval widths. This achievement owes largely to the stable time series feature extraction capabilities of the N-HiTS model and the remarkable flexibility and adaptability of the Gaussian likelihood distribution. In comparison to the DeepAR model, the proposed model exhibits satisfactory interval forecasting performance, with CWC improvements of 0.012, 0.111, and 0.302 for 1, 3, and 6-step forecasting, respectively. Even amidst high data volatility scenarios, this model accurately tracks the evolving trends of ST.

### 3.6 Performance analysis of feature selection method

Feature selection has the capability to enhance the predictive accuracy of models and reduce their training duration. To elucidate the efficacy of feature selection in optimizing the performance of forecasting models, we inputted different feature combinations obtained from the importance ranking in Experiment 3.2 into the SP-N-HiTS-G model for training and testing in 6-step forecasting task. The results from **Table 7** show that the SP-N-HiTS-G model achieves optimal predictive performance with three features, validating the accuracy of the feature selection outcomes in Experiment 3.2. After feature selection, the SP-N-HiTS-G forecasting shows respective increases of 4.3%, 4.1%, and 11.6% in RMSE, MAE, and CWC, compared to directly inputting all features. In conclusion, the feature selection method proposed in this study proves to be efficient.

**Table 7:** Comparative analysis of forecasting performance of different feature combinations.

Feature number	RMSE	MAE	MAPE	PICP	PINRW	CWC
1	0.457	0.298	1.182	0.803	0.142	0.090
2	0.418	0.248	0.985	0.805	0.125	0.082
**3**	**0.393**	**0.243**	**0.973**	0.801	0.116	**0.077**
4	0.397	0.247	0.977	0.800	0.120	0.080
5	0.420	0.251	1.004	0.801	0.118	0.077
6	0.425	0.257	1.036	0.808	0.125	0.082
7	0.411	0.253	1.017	0.806	0.133	0.086

The bold represent superior performance.

### 3.7 Comparison of different parameter search methods

**Figure 11:**
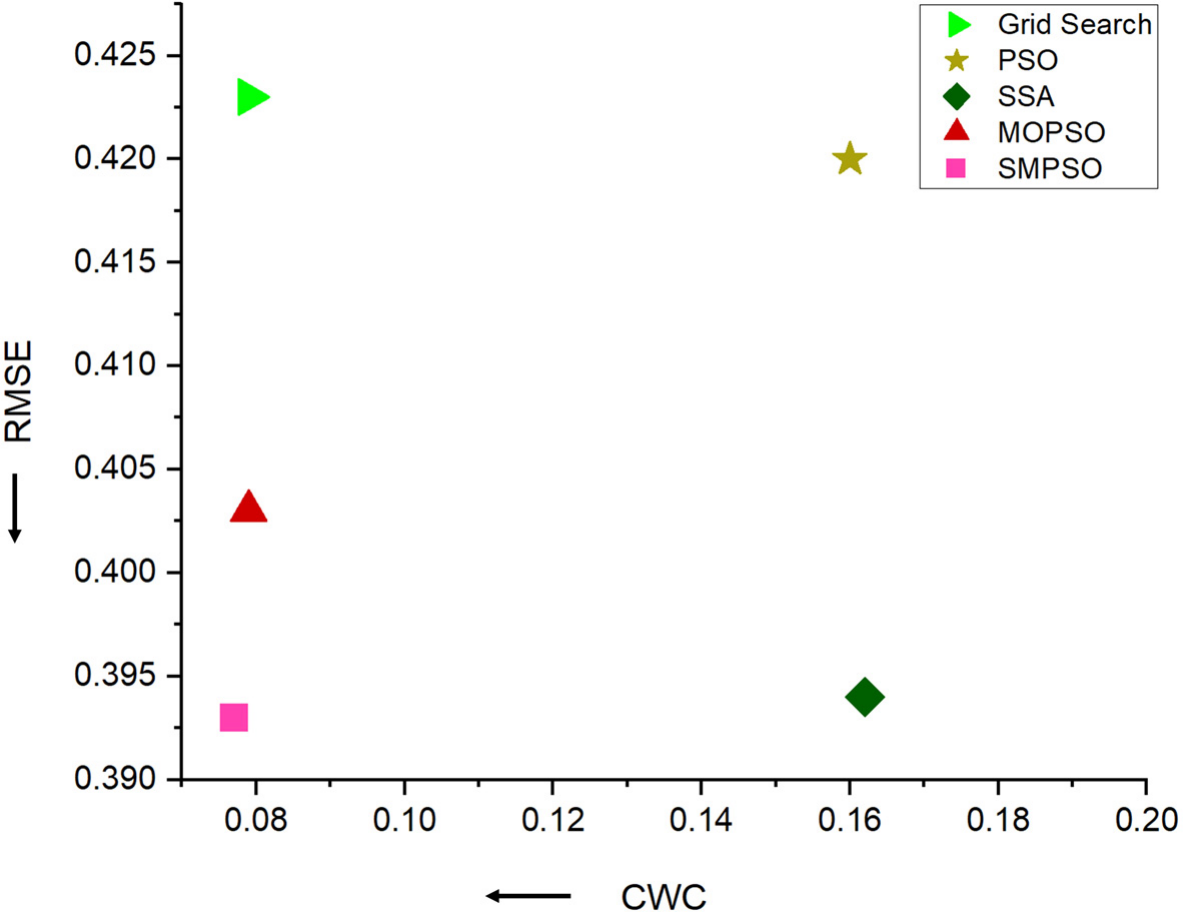
Forecasting performance results of different parameter search methods on N-HiTS-G.

To assess the effectiveness of the SMPSO algorithm, we conducted three sets of comparative experiments. The first set used grid search, the second set applied the single-objective optimization algorithms PSO and SSA, and the third set involved the multi-objective optimization algorithms MOPSO and SMPSO. In the case of single-objective optimization, as only one objective could be optimized, we chose the point forecast metrics MAE as the optimization goal. Conversely, the multi-objective optimization algorithms employed both the point forecast metrics MAE and the interval forecast metric CWC as optimization objectives. The results, as shown in **Table 8** and **Figure 11**, reveal the following:

The grid search method produces a balanced model that ensures good point forecast performance while also considering interval forecast performance, making it a reliable method, although it requires a significant amount of time. However, its overall performance is not the best.
Single-objective optimization algorithms have the potential to enhance model point forecast performance. However, overall, the models optimized by PSO and SSA generate inadequate forecasting intervals, evidenced by high CWC metrics. This suggests that single-objective optimization algorithms fail to consider both point and interval forecast performances simultaneously, although it reduces the parameter search time.
Multi-objective optimization algorithms not only optimize the parameter search time but also ensure both accuracy and stability in point and interval forecasting. Moreover, compared to the MOPSO algorithm, the forecasting model optimized by SMPSO achieves the best overall results in both point and interval forecasts. This indicates that the SMPSO algorithm has superior and stable parameter search capabilities, making it highly practical for early warning and dynamic regulation of soil conditions.

**Table 8:** Comparative analysis of forecasting performance of different parameter search methods.

Method	RMSE	MAE	MAPE	PICP	PINRW	CWC
Grid Search	0.423	0.256	1.029	0.805	0.121	0.079
PSO	0.420	0.254	1.016	0.784	0.113	0.160
SSA	0.394	**0.243**	**0.973**	0.782	0.114	0.162
MOPSO	0.403	0.249	0.994	0.803	0.120	0.079
**SMPSO**	**0.393**	**0.243**	**0.973**	0.801	0.116	**0.077**

The bold represent superior performance.

## 4 Conclusion

Foliage plants are crucial for promoting urban greening and agricultural economic development. These plants have high requirements for environmental conditions, and a suitable soil temperature range can effectively promote their growth and development, ensuring stable yield and excellent ornamental value. Given the coupling, non-linearity, and complexity of soil temperature variations, this study proposes a rapid and efficient multivariate forecasting method, which can accurately predict soil temperature trends for the next 20, 60, and 120 minutes. Experimental results show that our proposed multivariate, multi-step forecasting model based on SP-N-HiTS-G outperforms other models, demonstrating both superior accuracy and enhanced stability in forecasting performance. The key research content is as follows:

To address the issue of high-dimensional data, this study employs Gradient Boosting Tree models as feature selectors to identify important environmental factors related to soil temperature. After feature selection, the proposed model achieved reductions in RMSE, MAE, and CWC by 0.018, 0.010, and 0.044% respectively for forecasts up to 120 minutes, demonstrating that this feature selection method can effectively extract important environmental factors, reducing model training time while enhancing predictive accuracy.
This study established a novel forecasting model based on N-HiTS-G, which combines the N-HiTS model with a Gaussian likelihood function. The model can accurately forecast future soil temperature trends, with a stable inference speed of around 20 seconds. Compared to commonly used or advanced benchmark models, this model offers higher predictive accuracy and faster inference speeds. Furthermore, it produces reliable forecasting intervals, effectively reducing the uncertainty of multi-step forecasting.
This study employs the multi-objective optimization algorithm SMPSO to address the parameter determination problem in the forecasting model. Compared to grid searching or manual tuning, this method significantly reduces the labor and time costs associated with determining model parameters. Moreover, compared to single-objective optimization algorithms, multi-objective optimization can train more precise and stable models. The optimized SP-N-HiTS-G model provides forecasts with MAE values of 0.057, 0.136, and 0.241 for 20, 60, and 120 minutes into the future respectively, accompanied by outstanding interval forecasting performance, making it more suitable for facility cultivation environment applications.

This study provides guidance for important issues related to environmental optimization in facility cultivation, helping to optimize cultivation conditions, improve plant growth efficiency, thus promoting the development of sustainable agriculture. Moreover, this study also contributes to the application of artificial intelligence technology in smart agriculture scenarios, such as agricultural intelligent monitoring systems (Chamara et al., 2022), multi-modal environmental monitoring that integrates visual and temporal technologies (Li et al., 2024), and the use of smart agricultural robots (Roldán et al., 2018). Our model not only achieves accurate and reliable multi-step interval forecasting but can also be widely applied in edge-side inference for intelligent equipment, environmental optimization, and energy conservation, demonstrating higher practical value in real-world facility cultivation environments. In the future, we hope to further refine our approach. Specifically, We will continue to apply methods for real-time model updates under facility cultivation, as well as conduct research on accurate forecasting models for longer forecasting periods.

## Data Availability

The raw data supporting the conclusions of this article will be made available by the authors, without undue reservation.
